# Mitigating selection bias in organ allocation models

**DOI:** 10.1186/s12874-021-01379-7

**Published:** 2021-09-21

**Authors:** Erin M. Schnellinger, Edward Cantu, Michael O. Harhay, Douglas E. Schaubel, Stephen E. Kimmel, Alisa J. Stephens-Shields

**Affiliations:** 1 Department of Biostatistics, Epidemiology and Informatics, Perelman School of Medicine, University of Pennsylvania, 423 Guardian Drive, Blockley Hall Room 107, Philadelphia, PA 19104 USA; 2 Department of Surgery, Division of Cardiovascular Surgery, Hospital of the University of Pennsylvania, Philadelphia, PA USA; 3 Department of Epidemiology, College of Public Health and Health Professions & College of Medicine, University of Florida, Gainesville, FL USA

**Keywords:** Selection bias, Survivor bias, Transplant benefit, Waitlist urgency, Organ allocation

## Abstract

**Background:**

The lung allocation system in the U.S. prioritizes lung transplant candidates based on estimated pre- and post-transplant survival via the Lung Allocation Scores (LAS). However, these models do not account for selection bias, which results from individuals being removed from the waitlist due to receipt of transplant, as well as transplanted individuals necessarily having survived long enough to receive a transplant. Such selection biases lead to inaccurate predictions.

**Methods:**

We used a weighted estimation strategy to account for selection bias in the pre- and post-transplant models used to calculate the LAS. We then created a modified LAS using these weights, and compared its performance to that of the existing LAS via time-dependent receiver operating characteristic (ROC) curves, calibration curves, and Bland-Altman plots.

**Results:**

The modified LAS exhibited better discrimination and calibration than the existing LAS, and led to changes in patient prioritization.

**Conclusions:**

Our approach to addressing selection bias is intuitive and can be applied to any organ allocation system that prioritizes patients based on estimated pre- and post-transplant survival. This work is especially relevant to current efforts to ensure more equitable distribution of organs.

**Supplementary Information:**

The online version contains supplementary material available at 10.1186/s12874-021-01379-7.

## Background

The Organ Procurement and Transplantation Network (OPTN) is responsible for allocating deceased-donor organs in the United States. The OPTN utilizes separate policies to govern the allocation of livers, kidneys, hearts, and lungs [1]. For example, liver transplant candidates receive a Model for End-Stage Liver Disease (MELD) score; those wait-listed for kidney transplantation receive an Estimated Post-Transplant Survival (EPTS) score, and lung transplant candidates receive a Lung Allocation Score (LAS) [1–3]. More specifically, the LAS is derived from models that predict both pre-transplant and post-transplant survival and aims to balance each patient’s predicted transplant benefit (i.e., difference between survival with versus without a lung transplant) against their waitlist urgency [1–3].

Such models are particularly susceptible to selection bias. Estimates of pre-transplant survival are subject to selection bias in the form of dependent censoring, because patients can be removed from the waiting list prior to 1 year of follow-up due to receipt of transplant, loss to follow-up, or other clinical reasons (e.g., the inability to withstand the transplant surgery). In each of these cases, the patients’ true one-year pre-transplant survival is unobserved. Estimates of post-transplant survival similarly are subject to a type of selection bias – “survivor bias” [4–7] – because they have been derived using information only among patients who received a transplant. Thus, post-transplant survival models are applied to all wait-listed patients, but are fitted using only transplanted patients. In particular, while statistical models used to estimate transplant benefit account for variation in patient characteristics, they do not account for the fact that in order to receive a transplant, individuals must: 1) survive on the waitlist long enough for a suitable donor organ to become available, and 2) have sufficient priority to actually receive the organ. Since individuals who survive 1 year or more on the waitlist might be inherently different from individuals who die, receive a transplant, or are censored (e.g., lost to follow-up or removed from the waitlist for other clinical reasons, such as being too sick to withstand the transplant surgery) prior to 1 year, failure to incorporate such information in the models used to estimate transplant benefit and waitlist urgency can lead to inaccurate predictions.

Lung transplantation represents an important example to study because it is a highly effective treatment, but organs are scarce. Waitlist mortality is high, waitlist times vary, and there are concerns about inequities in waitlist mortality and organ allocation [8]. In fact, concerns about these inequities led the Department of Health and Human Services to mandate the development of the LAS based on medical need rather than wait time [3, 9–11]. Donor lungs are now allocated to recipients based on the LAS [3, 9–11] which is calculated using the predicted difference between transplant benefit and waitlist urgency, with transplant benefit defined as one-year post-transplant survival minus one-year waitlist survival, and waitlist urgency defined as one-year waitlist survival. Conceptually, the LAS aims to determine the number of days of life a person would gain over the next year if they receive transplant compared to if they do not receive transplant, and prioritizes patients for whom this comparison is more favorable.

In Appendix 1, we use directed acyclic graphs to illustrate how selection bias can lead to inaccurate predictions for both the pre-transplant and post-transplant prediction models. This bias can occur even with measured covariates, because: 1) not all measured covariates which are available in the UNOS database are included in the LAS models (e.g., geographic differences in transplant listing and outcomes [12]); and 2) even if a measured covariate is included in the LAS, the association between such a covariate and post-transplant survival can be different in the post-transplant subset than it is in the full waitlist population. Biased estimates of pre- and post-transplant survival in turn imply that the current prioritization of lung transplant recipients may be inaccurate. Although prior research has incorporated weights in the pre-transplant survival model [13], the models did not capture important geographic differences in patient selection and survival, and no work, to our knowledge, has estimated weights to the post-transplant survival model.

In this study, we attempt to bring principles from causal inference into the existing prediction model framework employed by the LAS to improve organ allocation. Specifically, we develop a modified LAS using inverse probability weighting to improve the accuracy of the LAS by accounting for selection bias in the pre- and post-transplant survival models. Our work incorporates additional factors in the pre-transplant weights to better address selection bias in the pre-transplant survival model. We also develop new weights to address selection bias in the post-transplant survival model.

## Methods

### Data & Weights

We used publicly available pre- and post-lung transplant data from the United Network for Organ Sharing (UNOS). Our development cohort consisted of all patients 18 years or older who were listed for single or bilateral lung transplantation in the United States between January 1, 2010 and December 31, 2013. This date range was chosen because it ensures that no patient experiences any person-time prior to the implementation of the LAS (i.e., prior to May 2005) and is consistent with the development cohort used to fit the current LAS models. Our testing cohort consisted of patients meeting these same criteria who were listed between January 1, 2016 and December 31, 2017 (the last complete year of available data). Patients listed during 2014 and 2015 were excluded from our analyses to ensure that 1) our development cohort is consistent with the development cohort used to fit the current LAS; and 2) our testing cohort does not include any patients for whom prior versions of the LAS were used in clinical practice. To avoid concerns about positivity violations associated with the likelihood of receiving a transplant, we removed individuals who had clinical contraindications to receiving transplant (e.g., those with panel reactive antibodies greater than 90%), and individuals with both restrictive lung disease (diagnosis group D) and height less than five feet who require such small donor organs that they rarely find a match. In both cohorts, patients were followed from their initial listing date to their time of transplant, death, loss to follow-up, or removal from the waitlist due to other clinical reasons (e.g., inability to withstand the transplant surgery), whichever occurred first. Appendix 2 provides demographic and clinical characteristics of the development and testing cohorts.

After cleaning (see Appendix 3), data were divided into pre- and post-transplant subsets, with the pre-transplant subset containing daily time intervals, and the post-transplant subset consisting of a single record per patient. This data structure allowed us to construct time-varying inverse probability of treatment weights (IPTW) and inverse probability of censoring weights (IPCW), which effectively circumvent survivor bias by “mapping” the survival probabilities obtained among the post-transplant group back to the full waitlist population. Appendix 4 and 5 provide methodologic details on how we constructed these weights. Analyses were conducted using Stata (StataCorp LLC, College Station, TX) and R (R Foundation for Statistical Computing, Vienna, Austria).

### Fitting the outcome models

We fit a weighted Cox proportional hazards model to each patient’s baseline record in the pre-transplant data, weighted by each patient’s daily time-varying weight to estimate one-year pre-transplant survival accounting for dependent censoring [14]. This approach allows us to incorporate information from time-varying covariates and time on the waitlist captured by the IPTW and IPCW models, while still retaining the same form of the outcome model as the current pre-transplant LAS. It also provides predicted probabilities of one-year survival on the waitlist, which is consistent with the definition of waitlist urgency used by the current LAS. Our weighted Cox proportional hazards model contains all covariates included in the existing pre-transplant LAS (but not follow-up time, as UNOS policy prohibits follow-up time from being included in the outcome model). Thus, the variables in the outcome model are the same in the modified LAS as in the existing LAS, but the coefficient estimates vary (see Appendix 6).

Similarly, we estimated one-year post-transplant survival by fitting a weighted Cox proportional hazards model to the post-transplant subset which included the covariates in the existing post-transplant LAS, and was weighted by each patient’s post-transplant weight (which is fixed at the time of transplant). Consistent with UNOS policy, the post-transplant outcome model did not include time since waitlist registration. The estimate of one-year post-transplant survival obtained from this weighted outcome model differs from that included in the current LAS, as the weighted outcome model provides an estimate of survival that reflects the *entire* waitlist population, whereas the current LAS estimates this quantity only among the subset of individuals who did, in fact, receive transplant. Taken together, the weighted pre- and post-transplant outcome models produce survival estimates that align more closely with the LAS’s conceptual goal of comparing the number of days of life a person would gain over the next year if they receive transplant versus if they do not receive transplant.

### Assessing model performance

To assess the discrimination of the pre- and post-transplant outcome models, we constructed time-dependent receiver operating characteristic (ROC) curves and evaluated the area under these curves (AUC) via nearest-neighbor smoothing at 1 year post-waitlist registration and 1 year post-transplantation, respectively [15, 16]. This approach accommodates censoring by viewing survival time as a “time-varying binary outcome” at each possible time point, and estimating the sensitivity and specificity of the model among all patients who are still alive and at risk of the outcome at those time points [15, 16]. Separate statistics were computed for the development and testing cohorts.

Calibration was evaluated graphically by defining low-, medium-, and high-risk categories based on tertiles of the linear predictor of the pre- and post-transplant outcome models, averaging the survival functions within each of these risk categories, and then overlaying the observed (Kaplan-Meier) and predicted survival curves for each risk category [17]. This approach allows us to evaluate the calibration of the pre- and post-transplant outcome models at any time point after waiting list registration among all patients who are alive and at risk of the outcome at those time points [17]. Separate calibration plots were constructed for the development and testing cohorts.

### Comparing the modified LAS to the existing LAS

The current LAS is composed of a pre-transplant outcome model and a post-transplant outcome model, where only the pre-transplant outcome model is weighted using a select number of covariates [13]. These outcome models are used to predict one-year waitlist and one-year post-transplant survival, which are combined into a raw score by computing one-year post-transplant survival minus two times one-year waitlist survival [1, 3, 11]. This raw score is normalized so that it ranges from 0 to 100, with higher values indicating greater priority for transplantation [1, 3, 11]. We construct modified pre- and post-transplant outcome models by applying weights to both models, as described above. We applied the weighted pre- and post-transplant outcome models to the testing cohort to estimate a modified LAS score for each patient considering all possible offer dates in 2016 and 2017. At each offer date, we subset the data to include only patients who were alive, registered on the waitlist, and not yet transplanted at that date, as per UNOS guidelines [1, 11]. We computed daily, person-specific survival estimates for the first year spent on the waitlist and the first year post-transplant using the baseline hazard, weighted model coefficients, and each individual’s covariate values. Each patients’ resulting waitlist and post-transplant survival probabilities were summed to obtain the modified waitlist urgency and modified post-transplant survival measures (further details in Appendix 7).

Existing LAS scores were estimated for each patient by applying the published pre- and post-transplant LAS models to the testing cohort following the same procedure as above [1, 11] with coefficients from the existing LAS. Last, we constructed two sets of rankings for eligible patients at each offer date: rankings based on their modified LAS scores and rankings for the same patients based on their existing LAS scores.

To assess the difference between the modified and existing LAS models, we constructed Bland-Altman plots of 1) the modified LAS score versus the existing LAS score; and 2) the modified patient rank versus the existing patient rank [18]. We also created a scatter plot of the difference in estimated post-transplant survival versus the difference in pre-transplant survival obtained under the modified and existing LAS models to examine which of these factors drive changes in patient prioritization.

## Results

The development and testing cohorts were comparable in terms of demographic and clinical characteristics (Appendix 2). Table 1 displays the time-dependent AUC [15, 16] evaluated at 1 year post-waitlist registration and 1 year post-transplant for the modified and existing LAS models in the development and testing cohorts. In all cases, the AUC of the modified model is higher than that of the existing LAS, indicating that the modified model has better discrimination. However, the extent of improvement is larger in the pre-transplant population than in the post-transplant population.

**Table 1:** Time-dependent AUC estimated via nearest-neighbor smoothing [15, 16] at one year post-waitlist registration and one year post-transplant for the modified and existing LAS models. 95% confidence intervals were computed via the bootstrap percentile method; *p*-values were estimated using the normal approximation and standard deviation obtained from the bootstrap replicates. 1000 bootstrap replicates were used

Cohort^a^		Modified LAS	Existing LAS	Difference	*p*-value of difference
Development	Pre-tx	0.732 (0.690, 0.760)	0.660 (0.619, 0.697)	0.071 (0.030, 0.106)	< 0.001
	Post-tx	0.605 (0.580, 0.629)	0.560 (0.531, 0.585)	0.045 (0.026, 0.065)	< 0.001
Testing	Pre-tx	0.750 (0.686, 0.792)	0.693 (0.631, 0.738)	0.057 (−0.004, 0.122)	0.083
	Post-tx	0.570 (0.536, 0.606)	0.540 (0.507, 0.576)	0.030 (0.003, 0.058)	0.030

^a^*tx:* transplant

**Fig. 1:**
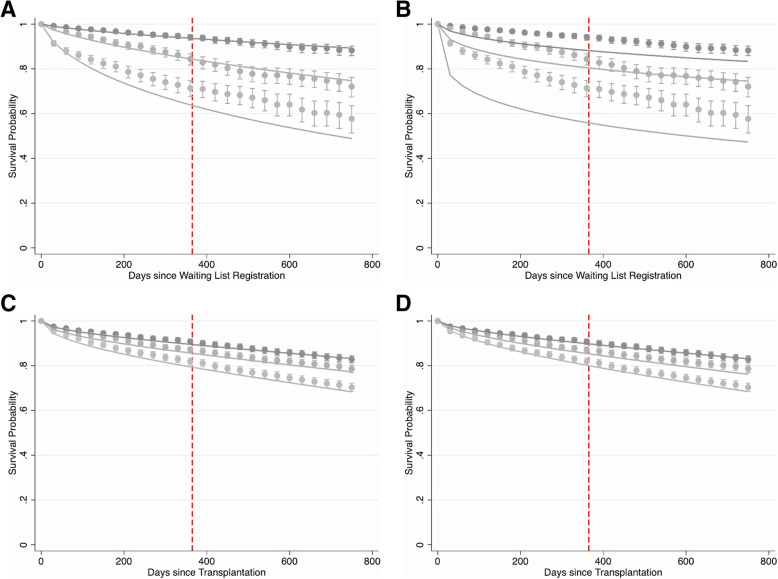
Observed vs. Predicted Survival in Development Cohort. Time-dependent calibration of A) the modified pre-transplant outcome model, B) the existing pre-transplant LAS model, C) the modified post-transplant outcome model, and D) the existing post- transplant LAS model, in the development cohort. Smooth, solid lines represent predicted survival probabilities; points with vertical error bars represent observed Kaplan-Meier estimates with their corresponding 95% confidence intervals. Estimates were plotted every 30 days to ease plot readability. Three risk groups are shown: low-risk/best survival (darkest lines), medium-risk/intermediate survival (medium-shaded lines), and high-risk/worst survival (lightest lines). A vertical, dashed, red line is placed at one year post-waitlist registration for reference

**Fig. 2:**
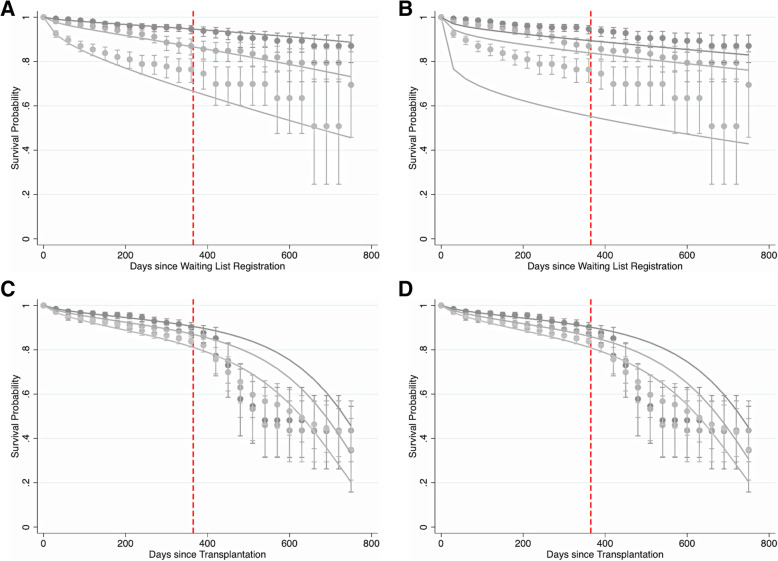
Observed vs. Predicted Survival in Testing Cohort. Time-dependent calibration of A) the modified pre-transplant outcome model, B) the existing pre-transplant LAS model, C) the modified post-transplant outcome model, and D) the existing post-transplant LAS model, in the testing cohort. Smooth, solid lines represent predicted survival probabilities; points with vertical error bars represent observed Kaplan-Meier estimates with their corresponding 95% confidence intervals. Estimates were plotted every 30 days to ease plot readability. Three risk groups are shown: low-risk/best survival (darkest lines), medium-risk/intermediate survival (medium-shaded lines), and high-risk/worst survival (lightest lines). A vertical, dashed, red line is placed at one year post-waitlist registration for reference

Figure 1 depicts the time-dependent calibration of the modified pre- and post-transplant outcome models and existing pre- and post-transplant LAS models in the development cohort for the first 2 years post-listing and post-transplant. The predicted survival curves from the modified pre-transplant outcome model agree more closely with the observed survival curves for all three risk categories when compared with the existing LAS model. Conversely, predictions from the existing pre-transplant LAS model are noticeably different from the observed survival curves (Fig. 1B). This discrepancy is most prominent during the first year post-waitlist registration, but continues beyond this time point for all three risk groups (Appendix 8). In contrast, predicted survival estimates from the modified post-transplant outcome model (Fig. 1C) and the existing post-transplant LAS model (Fig. 1D) closely match the observed survival curves. This observation is consistent with the AUC results in Table 1, and suggests that the extent of improvement in calibration is more noticeable for the pre-transplant model than the post-transplant model. Similar results were obtained in the testing cohort (Fig. 2). In the testing cohort, the predicted survival estimates from the modified pre-transplant outcome model were consistent with the observed survival curves over time, regardless of risk group (Fig. 2A). Predictions obtained from the existing pre-transplant LAS model, however, did not align as well with observed survival (Fig. 2B). The modified and existing post-transplant models exhibit similar calibration in the testing cohort (Fig. 2C and D, respectively). The calibration of both models is quite good in the first year post-transplant but deteriorates considerably beyond 1 year for all three risk groups (Appendix 8).

**Fig. 3:**
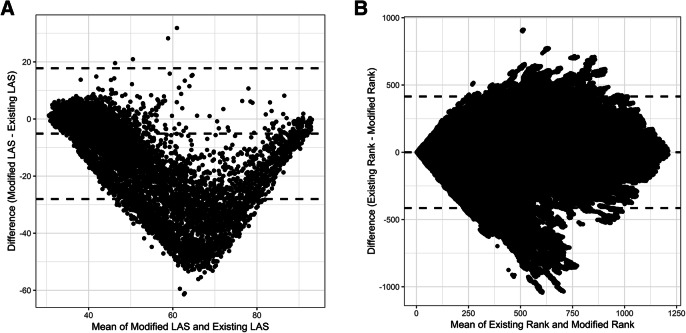
Bland-Altman plots. A) Difference between the modified and existing LAS scores versus the mean of the two scores, and B) difference between the modified and existing patient ranks versus the mean of the two ranks, from 712 different organ offer dates in the testing cohort. Horizontal, dashed lines are placed at ±2 standard deviations of the mean

Figure 3 depicts Bland-Altman plots of A) the modified versus existing LAS score, and B) the modified versus existing patient rank, for 712 different organ offer dates in the testing cohort. Patients at the extremes tend to receive similar scores under the two models, while patients with intermediate scores tend to experience more changes under the modified LAS. Specifically, a distinct cluster of patients appears more than 2 standard deviations below the mean; for these patients, the modified LAS predicts a lower score than the existing LAS does. The Bland-Altman plot of changes in rank (Fig. 3B) exhibits a somewhat different pattern than that in Fig. 3A due to constraints of ranks (i.e., individuals who receive a more favorable rank are necessarily balanced by those who receive a less favorable one).

**Fig. 4:**
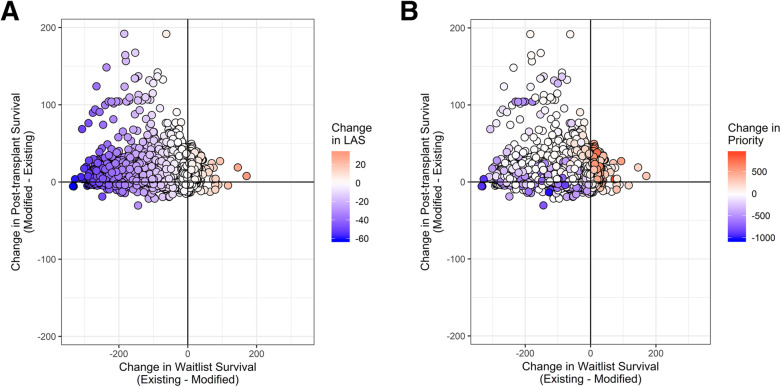
Scatter plots of the difference in post-transplant survival versus the difference in waitlist survival obtained under the modified and existing LAS models for 712 organ offer dates in the testing cohort. Points represent patients, and are shaded based on the magnitude of change in A) score, with red representing increases in score and blue representing decreases in score; or B) rank, with red representing higher (better) priority and blue representing lower (worse) priority

Figure 4 illustrates how differences in estimated pre- and post-transplant survival under the modified and existing LAS models influence patients’ LAS scores (Fig. 4A) and prioritization (Fig. 4B) for 712 organ offer dates in the testing cohort. Consistent with Fig. 3A, the majority of patients in Fig. 4A are shaded white or blue, indicating that these patients would receive the same or lower score under the modified model compared with the existing LAS. This lower score does not always translate into a lower (worse) priority, because the rank of a particular patient on a particular organ offer date depends on the ranks of all other eligible patients on the waitlist at that date. Consequently, the distribution of shading in Fig. 4B differs from that in Fig. 4A. In both figures, however, differences in pre-transplant survival explain a greater proportion of the variability in the outcome (i.e., change in LAS score or change in priority) compared to differences in post-transplant survival.

## Discussion

We developed a weighted estimation strategy to account for selection bias in the pre- and post-transplant models used to calculate LAS scores in prioritizing patients for lung transplant. To our knowledge, we are the first to incorporate weighting into the fitting of post-transplant models to account for survivor bias and other forms of selection into the post-transplant population. We also improve upon weighted fitting of pre-transplant models by incorporating additional variables in our weight models to better account for dependent censoring – most notably, geography. Since these variables are only included in the weight models (not the outcome models), they would only influence the performance of the outcome model if they are associated both with survival and with patients’ selection for transplantation. The fact that discrimination and calibration improve under the modified pre- and post-transplant outcome models compared to the existing LAS models suggests that regional differences in patient selection may be important to consider when estimating pre- and post-transplant survival. That said, the extent of improvement is larger in the pre-transplant population (i.e., the full waiting list population) than in the post-transplant subset. There are two potential explanations for this observation: first, we have considerably more follow-up time in the pre-transplant population than in the post-transplant population; and second, selection bias appeared to have a larger impact on the estimate of waiting list urgency than on the estimate of post-transplant survival.

We postulated that the current LAS underestimates predicted transplant benefit due to survivor bias because it only predicts this quantity among people who were indeed selected to receive transplant, who tend to be older and sicker. Our results (e.g., Fig. 4A and B) are consistent with this idea because they suggest that patients’ estimated post-transplant survival under the modified LAS would be the same or greater than their estimated post-transplant survival under the existing LAS. Because the estimate of pre-transplant survival also tends to be longer under the modified LAS compared to the existing LAS, a sizable number of patients with intermediate scores under the current LAS would receive lower scores (lower priority) under the modified LAS.

Our study has several strengths. We demonstrate how inverse probability weighting can account for survivor bias in real-world lung transplant data. These weights account for the probability of receiving transplant as well as the probability of censoring. By including additional variables in our weighting, such as geography and prior (i.e., lagged) clinical covariates, we account for regional variability in pre- and post-transplant survival and are able to capture patients’ waitlist history and clinical trajectory more fully, while maintaining consistency between our modified outcome model and the existing LAS. This approach ensures a fair comparison between the modified and existing models in both the development and testing cohorts. Although our primary endpoints were one-year pre- and post-transplant survival, the results using greater extent of follow-up time demonstrate how these models perform over longer time frames. Such an evaluation can help inform future revisions of the LAS, and is especially relevant as the lung transplant community considers what role longer-term survival should play in lung allocation [19–21]. Finally, while our analysis focuses on lung allocation specifically, our approach can be applied to any organ allocation system that relies on estimates of post-transplant survival to prioritize patients (e.g., the United States’ EPTS score for kidney allocation [1–3], Germany’s Lung Allocation Score [9, 22], and the United Kingdom’s Liver Transplant Benefit Score [23]).

Our study is not without limitations. First, insufficient information is available to distinguish between patients who were newly listed and those who were re-activated after temporary waitlist removal. Thus, the first record associated with each identification number was taken to be the initial registration date, follow-up time was counted from that date forward, and individuals who were subsequently lost to follow-up were censored at that time. Second, we exclude individuals on the waitlist who are highly unlikely to receive transplant, due to certain patient characteristics that prevent them from finding a suitable donor organ match (e.g., high sensitization or small stature). Although this analytic decision ensures that the remaining individuals on the waitlist have at least some probability of receiving transplant, it also implies that we can only generalize our findings to individuals on the waitlist who do not have these clinical contraindications. Third, we cannot account for ascertainment bias/informed presence bias (i.e., we cannot account for the fact that presence in the UNOS database is not random, but rather indicates that the patient was ill enough to visit the hospital, undergo evaluation for transplant, and be registered on the waitlist). Fourth, transplant organ allocation is a highly selective process, and selection bias can occur at various stages throughout this process (e.g., decision to register a patient on the waitlist, decision to remove a waitlisted patient, decision to transplant). In this particular paper, we restrict our focus to selection bias that arises due to the fact that some individuals die or are otherwise censored prior to receiving transplant, and present a quantitative approach to mitigating this bias in the LAS. Although beyond the scope of this study, additional research – including qualitative work – is necessary to understand how to balance all the factors that go into making transplant decisions.

## Conclusions

Our approach to addressing selection bias is intuitive and straightforward to implement, and demonstrates how principles from causal inference can be incorporated into existing prediction model frameworks to improve organ allocation. Additionally, it can be applied to any organ allocation system that relies on estimates of pre- and post-transplant survival to prioritize patients, including those used for different organs and in other countries. We anticipate that this work can inform future revisions of the LAS and other prediction models in organ transplantation to improve prediction and ensure fair and equitable organ allocation.

## Supplementary Information

**Additional file 1.**

**Additional file 2.**

## Data Availability

This data that support the findings of this study are available from the United Network for Organ Sharing (UNOS). The authors do not have the authority to share UNOS data; researchers interested in accessing this data must submit a request to UNOS directly. All code is available upon request to the corresponding author, Ms. Erin M. Schnellinger.

## Abbreviations

AUC: Area Under receiver operating characteristic Curve
EPTS: Estimated Post-Transplant Survival score
LAS: Lung Allocation Score
MELD: Model for End-Stage Liver Disease score
OPTN: Organ Procurement and Transplantation Network
ROC: Receiver Operating Characteristic curve
UNOS: United Network for Organ Sharing
