# Supplementary material for: Mitigating selection bias in organ allocation models
Source: BMC Med Res Methodol. 2021 Sep 21;21:191. doi: 10.1186/s12874-021-01379-7 (PMC8454078; doi:10.1186/s12874-021-01379-7)
Supplement: Supplementary file 1 — Additional file 1. [file 12874_2021_1379_MOESM1_ESM.docx]

**Mitigating Selection Bias in Organ Allocation Models**

**Supplementary Appendices**

Table of Contents

[APPENDIX 1: Directed Acyclic Graph (DAG) 2](#_Toc64642616)

[APPENDIX 2: Characteristics of the Development and Testing Cohorts 4](#_Toc64642617)

[APPENDIX 3: Data Cleaning 7](#_Toc64642618)

[APPENDIX 4: Constructing the IPTW and IPCW Weights 8](#_Toc64642619)

[APPENDIX 5: Weight Truncation 11](#_Toc64642620)

[APPENDIX 6: Parameter Estimates for Modified Outcome Models 11](#_Toc64642621)

[APPENDIX 7: Calculating the Modified LAS Score 17](#_Toc64642622)

[APPENDIX 8: Long-term Calibration of Post-Transplant Outcome Model 17](#_Toc64642623)

[REFERENCES 19](#_Toc64642624)

# APPENDIX 1: DIRECTED ACYCLIC GRAPH (DAG)

Appendix Figure 1A shows the hypothesized DAG of the relationship between patients’ covariates at the time of their hypothetical organ offer [L(0)], receipt of transplant, and post-transplant survival prior to weighting. Whether or not a particular individual will survive long enough to receive transplant likely depends on post-baseline characteristics [L(t)], such as acute exacerbations of their illness and the amount of time they have already spent on the waitlist. These variables are also likely to influence how long the patient survives post-transplant. Before weighting, these variables are unaccounted for. At first, failure to account for these variables might seem fine, as Transplant is a collider on the path: Covariates informing LAS 🡪 Transplant 🡨 L(t) 🡪 Post-transplant survival. However, the patients who survive long enough to receive transplant may differ from those who do not. Moreover, the estimate of post-transplant survival used by the current LAS is inherently restricted to the former individuals. Thus, a spurious association is induced between patients’ covariates at the time of their hypothetical organ offer and post-transplant survival. This spurious association can lead to biased estimates of post-transplant survival, which implies that the current prioritization of lung transplant recipients may be inaccurate.

Appendix Figure 1B shows the same relationship between patients’ covariates at the time of their hypothetical organ offer [L(0)], receipt of transplant, and post-transplant survival after weighting. Essentially, the IPTW and IPCW weights we propose in the main text capture the information contained in L (i.e., post-baseline covariates and time on the waitlist), thereby removing the arrows into transplant. This approach should mitigate the survivor bias problem.


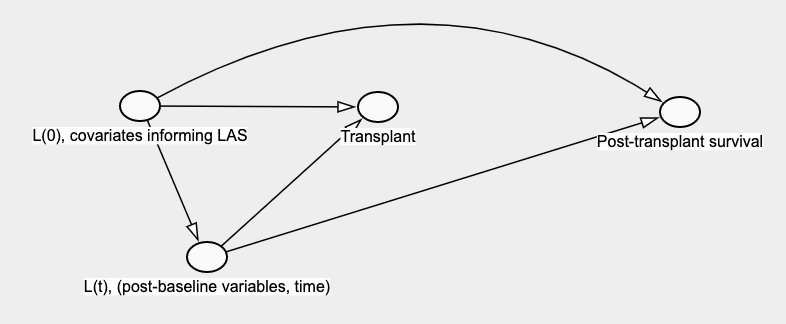
**A**


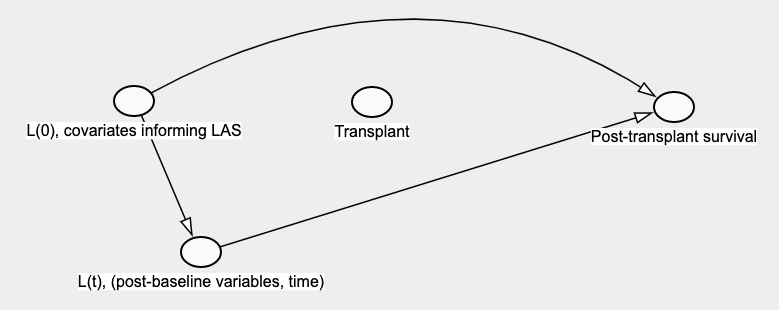


**B**

**Appendix Figure 1.** Hypothesized DAG depicting the relationship between patients’ covariates, receipt of transplant, and post-transplant survival A) prior to weighting, and B) after weighting.

# APPENDIX 2: CHARACTERISTICS OF THE DEVELOPMENT AND TESTING COHORTS

This study relies on pre- and post-lung transplant data from the United Network for Organ Sharing (UNOS). Our development cohort consisted of all patients 18 years or older who were listed for single or bi-lateral lung transplantation in the United States between January 1, 2010 and December 31, 2013. Our testing cohort consisted of patients meeting these same criteria who were listed between January 1, 2016 and December 31, 2017. Patients listed during 2014 and 2015 were excluded from our analyses to ensure that 1) our development cohort is consistent with the development cohort used to fit the current LAS; and 2) our testing cohort does not include any patients for whom prior versions of the LAS were used in clinical practice. To avoid concerns about positivity violations associated with the likelihood of receiving a transplant, we removed individuals who had clinical contraindications to receiving transplant (e.g., those with panel reactive antibodies greater than 90%), and individuals with both restrictive lung disease (diagnosis group D) and height less than five feet who require such small donor organs that they rarely find a match. In both cohorts, patients were followed from their initial listing date to their time of transplant, death, or loss to follow-up, whichever occurred first.

Appendix Table 1 displays demographic and clinical characteristics of the complete waiting list population in the development and testing cohorts; Appendix Table 2 displays similar information for the subset of patients in each cohort who received transplant. Overall, the full waiting list population is comparable in the development and testing cohorts. The subset of transplanted individuals are also comparable across cohorts. However, the amount of pre- and post-transplant follow-up time and the number of deaths (outcome events) are noticeably smaller in the testing cohort compared to the development cohort. This discrepancy is due to the fact that the UNOS data to which we have access was administratively censored in 2018. Thus, any pre- or post-transplant deaths that occurred beyond this date are not counted. As more follow-up time accrues, the number of pre- and post-transplant deaths in the testing cohort should increase, which will lead to increased precision in the observed Kaplan-Meier survival estimates shown in Figure 2 of the main text.

**Appendix Table 1: Demographic and clinical characteristics of the complete waiting list population in the development and testing cohorts.**

|  | **Development Cohort** | **Testing Cohort** |
| --- | --- | --- |
| Total number of patients (N) | 8379 | 5354 |
| Waiting time (days), median (IQR) | 73.0 (19.0, 237.0) | 57.0 (16.0, 154.0) |
| Death on waitlist within 1 year of waitlist registration | 56 (0.7%) | 25 (0.5%) |
| All deaths on waitlist | 751 (9.5%) | 288 (5.7%) |
| Removal from waitlist due to transplant | 7157 (85.4%) | 4154 (77.6%) |
| REGION |  |  |
| 1 | 251 (3.0%) | 185 (3.5%) |
| 2 | 1264 (15.1%) | 953 (17.8%) |
| 3 | 951 (11.3%) | 522 (9.7%) |
| 4 | 1216 (14.5%) | 636 (11.9%) |
| 5 | 1203 (14.4%) | 878 (16.4%) |
| 6 | 208 (2.5%) | 110 (2.1%) |
| 7 | 607 (7.2%) | 438 (8.2%) |
| 8 | 502 (6.0%) | 312 (5.8%) |
| 9 | 285 (3.4%) | 180 (3.4%) |
| 10 | 999 (11.9%) | 684 (12.8%) |
| 11 | 893 (10.7%) | 456 (8.5%) |
| GENDER |  |  |
| F | 3520 (42.0%) | 2263 (42.3%) |
| M | 4859 (58.0%) | 3091 (57.7%) |
| RACE/ETHNICITY |  |  |
| White | 6966 (83.1%) | 4224 (78.9%) |
| Black | 738 (8.8%) | 552 (10.3%) |
| Hispanic | 494 (5.9%) | 411 (7.7%) |
| Asian | 131 (1.6%) | 124 (2.3%) |
| Other | 50 (0.6%) | 43 (0.8%) |
| DIAGNOSIS |  |  |
| A (Obstructive Disease) | 2423 (28.9%) | 1444 (27.0%) |
| B (Pulmonary Hypertension) | 398 (4.7%) | 299 (5.6%) |
| C (Cystic Fibrosis) | 975 (11.6%) | 538 (10.0%) |
| D (Pulmonary Fibrosis) | 4583 (54.7%) | 3073 (57.4%) |
| BLOOD TYPE |  |  |
| A | 3297 (39.3%) | 2100 (39.2%) |
| AB | 307 (3.7%) | 195 (3.6%) |
| B | 916 (10.9%) | 607 (11.3%) |
| O | 3859 (46.1%) | 2452 (45.8%) |
| Age (years), median (IQR) | 59.0 (49.0, 65.0) | 60.0 (52.0, 66.0) |
| Bilirubin (mg/dL), median (IQR) | 0.7 (0.7, 0.7) | 0.7 (0.7, 0.7) |
| BMI (kg/m^3^), median (IQR) | 25.5 (21.6, 28.9) | 26.0 (22.0, 29.2) |
| Height (feet), median (IQR) | 5.6 (5.3, 5.8) | 5.6 (5.3, 5.8) |
| Cardiac index <2 L/min/m^2^ | 2096 (25.0%) | 896 (16.7%) |
| Central venous pressure (mmHg), median (IQR) | 5.0 (5.0, 8.0) | 5.0 (5.0, 8.0) |
| Continuous mechanical ventilation | 301 (3.6%) | 217 (4.1%) |
| Creatinine (serum) (mg/dL), median (IQR) | 0.8 (0.7, 1.0) | 0.8 (0.7, 1.0) |
| Diabetes | 1892 (22.6%) | 1074 (20.1%) |
| Forced vital capacity % predicted, median (IQR) | 48.0 (38.0, 61.0) | 48.0 (38.0, 62.0) |
| Functional status (none) | 1027 (12.3%) | 604 (11.3%) |
| Oxygen need at rest (L/min), median (IQR) | 3.0 (2.0, 6.0) | 4.0 (2.0, 6.0) |
| pCO_2_, median (IQR) | 42.0 (40.0, 49.0) | 43.0 (40.0, 50.0) |
| Pulmonary artery systolic pressure (mmHg), median (IQR) | 37.0 (30.0, 47.0) | 38.0 (31.0, 48.0) |
| Six-minute walk distance (feet), median (IQR) | 870.0 (550.0, 1176.0) | 880.5 (580.0, 1142.0) |

**Appendix Table 2: Demographic and clinical characteristics of the subset of individuals in the development and testing cohorts who received transplant.**

|  | **Development Cohort** | **Testing Cohort** |
| --- | --- | --- |
| Total number of patients (N) | 7074 | 4154 |
| Follow-up time post-transplant (days), median (IQR) | 1439.0 (721.0, 1834.0) | 199.0 (68.0, 365.0) |
| Death within 1 year of transplant | 935 (13.2%) | 346 (8.3%) |
| All deaths post-transplant | 3063 (43.7%) | 420 (10.9%) |
|  |  |  |
| REGION |  |  |
| 1 | 201 (2.8%) | 160 (3.9%) |
| 2 | 1068 (15.1%) | 680 (16.4%) |
| 3 | 784 (11.1%) | 405 (9.7%) |
| 4 | 1030 (14.6%) | 534 (12.9%) |
| 5 | 1031 (14.6%) | 675 (16.2%) |
| 6 | 174 (2.5%) | 67 (1.6%) |
| 7 | 503 (7.1%) | 334 (8.0%) |
| 8 | 432 (6.1%) | 251 (6.0%) |
| 9 | 241 (3.4%) | 126 (3.0%) |
| 10 | 794 (11.2%) | 528 (12.7%) |
| 11 | 816 (11.5%) | 394 (9.5%) |
| GENDER |  |  |
| F | 2780 (39.3%) | 1594 (38.4%) |
| M | 4294 (60.7%) | 2560 (61.6%) |
| RACE/ETHNICITY |  |  |
| White | 5938 (83.9%) | 3339 (80.4%) |
| Black | 581 (8.2%) | 397 (9.6%) |
| Hispanic | 405 (5.7%) | 299 (7.2%) |
| Asian | 107 (1.5%) | 93 (2.2%) |
| Other | 43 (0.6%) | 26 (0.6%) |
| DIAGNOSIS |  |  |
| A (Obstructive Disease) | 2792 (39.5%) | 1038 (25.0%) |
| B (Pulmonary Hypertension) | 265 (3.7%) | 182 (4.4%) |
| C (Cystic Fibrosis) | 764 (10.8%) | 436 (10.5%) |
| D (Pulmonary Fibrosis) | 3253 (46.0%) | 2498 (60.1%) |
| BLOOD TYPE |  |  |
| A | 2821 (39.6%) | 1702 (41.0%) |
| AB | 266 (3.7%) | 157 (3.8%) |
| B | 772 (10.8%) | 474 (11.4%) |
| O | 3272 (45.9%) | 1821 (43.8%) |
| Age (years), median (IQR) | 60.0 (50.0, 65.0) | 61.0 (52.0, 66.0) |
| Bilirubin (mg/dL), median (IQR) | 0.7 (0.7, 0.7) | 0.7 (0.7, 0.7) |
| BMI (kg/m^3^), median (IQR) | 25.4 (21.6, 28.8) | 25.9 (22.1, 29.0) |
| Height (feet), median (IQR) | 5.6 (5.3, 5.8) | 5.6 (5.3, 5.8) |
| Cardiac index <2 L/min/m^2^ | 1406 (19.9%) | 596 (14.3%) |
| Central venous pressure (mmHg), median (IQR) | 5.0 (5.0, 8.0) | 5.0 (5.0, 8.0) |
| Continuous mechanical ventilation | 457 (6.5%) | 320 (7.7%) |
| Creatinine (serum) (mg/dL), median (IQR) | 0.8 (0.7, 1.0) | 0.8 (0.7, 1.0) |
| Diabetes | 1725 (24.4%) | 971 (23.4%) |
| Forced vital capacity % predicted, median (IQR) | 45.0 (35.0, 57.0) | 45.0 (36.0, 58.0) |
| Functional status (none) | 414 (5.9%) | 209 (5.0%) |
| Oxygen need at rest (L/min), median (IQR) | 4.0 (3.0, 6.0) | 4.0 (2.5, 8.0) |
| pCO_2_, median (IQR) | 45.0 (40.0, 53.0) | 45.0 (40.0, 53.0) |
| Pulmonary artery systolic pressure (mmHg), median (IQR) | 38.0 (32.0, 48.0) | 38.0 (31.0, 49.0) |
| Six-minute walk distance (feet), median (IQR) | 774.0 (403.0, 1068.0) | 800.0 (445.0, 1059.0) |

# APPENDIX 3: DATA CLEANING

Our analysis uses daily time intervals. If individuals presented to clinic multiple times in one day, their last record in that day was retained; conversely, if individuals did not present to clinic in a given day, covariate information from their most recent visit was used (i.e., last observation carried forward, LOCF). The use of LOCF is consistent with UNOS guidelines [1]. Any values which remained missing (i.e., due to the absence of recent covariate values for a particular patient) were then filled in following the UNOS value substitution policy [1]. This policy stipulates that if any covariates remain missing after LOCF, then those values should be replaced with a default value, which represents a value either in the normal range for that covariate or which would yield the lowest LAS score for that patient. These default values are publicly available [1]. After data cleaning, 8379 patients provided 1,751,912 total records.

# APPENDIX 4: CONSTRUCTING THE IPTW AND IPCW WEIGHTS

To estimate waitlist urgency and transplant benefit, the LAS relies on two separate outcome models. The first estimates one-year waitlist (pre-transplant) survival, while the second estimates one-year post-transplant survival. Circumventing survivor bias requires us to “map” the survival probabilities obtained among the post-transplant group back to the full waitlist population. To do so, we constructed inverse probability of treatment weights (IPTW) and inverse probability of censoring weights (IPCW) to create a pseudo-population which reflects the characteristics of the full waitlist population, rather than just the subset of patients who were selected to receive transplant. This approach accounts for differences between the post-transplant subset and the full waitlist population that arise due to 1) measured covariates which are available in the UNOS database, but not included in the existing LAS models; and 2) measured covariates that are already included in the LAS, but which have a different effect on post-transplant survival in the post-transplant subset than they do in the full waitlist population; it does not account for differences due to unmeasured covariates.

We define the following notation: $A$ indicates exposure (receipt of transplant); $L$ represents covariates; the subscript $i$ denotes observations pertaining to the same subject; and the subscript $k$ denotes the day in which the exposure or covariates were observed. Thus, $A_{\mathrm{ki}}$ represents the observed exposure at day $k$ for subject $i$, and can take on values $a_{\mathrm{ki}}=1$ or $a_{\mathrm{ki}}=0$ for transplanted or not, respectively. Similarly, $L_{\mathrm{ki}}=l_{\mathrm{ki}}$ represents the observed covariate values at day $k$ for subject $i$. Overbars indicate exposure or covariate history. Thus, $\bar{A}_{\left( k-1 \right)i}$ represents the vector of exposure values for subject $i$ up through day $k-1$ (which, by design, will equal 0 up until the time subject $i$ receives transplant); similarly, $\bar{L}_{\mathrm{ki}}$ represents the vector of covariate values for subject $i$ up through day $k$.

Weights were considered separately for the pre- and post-transplant outcome models. The IPTW for the pre-transplant model accounts for time-varying covariate values and variable time of transplant by patient. Stabilized IPTW (${sw}_{i}$) were constructed as the cumulative product of the probability of receiving transplant at each day, conditional on individuals not having received transplant yet (observed through the prior day ($\bar{A}_{\left( k-1 \right)i}$)) and covariate history observed through the current day ($\bar{L}_{\mathrm{ki}}$):

$${sw}_{i}=\frac{\prod_{k=0}^{K} Pr[A_{\mathrm{ki}}=a_{\mathrm{ki}}|\bar{A}_{\left( k-1 \right)i}=\bar{0}]}{\prod_{k=0}^{K} Pr[A_{\mathrm{ki}}=a_{\mathrm{ki}}|\bar{A}_{\left( k-1 \right)i}=\bar{0},\bar{L}_{\mathrm{ki}}=\bar{l}_{\mathrm{ki}}]}$$

The denominator of the pre-transplant IPTW weights was estimated using pooled logistic regression, where predictors included the time-varying covariates of the published waitlist LAS model (i.e., age, bilirubin, body mass index, cardiac index, central venous pressure, continuous mechanical ventilation, serum creatinine, diabetes, diagnosis group, forced vital capacity, functional status, oxygen need at rest, partial pressure of carbon dioxide, pulmonary artery systolic pressure, six-minute walk distance), and also blood type, gender, race, and height (consistent with [2]). Additional predictors included geography (to account for differences in survival across UNOS regions), time (days) since waitlist registration (modeled using restricted cubic splines with five knots; knots were chosen using Harrell’s recommended percentiles (i.e., 5, 27.5, 50, 72.5, and 95), as implemented by STATA’s *mkspline* function [3]), and one-month lagged versions of the covariates of the published waitlist LAS (to capture patients’ waitlist history). The numerator of the pre-transplant IPTW weights was also estimated using pooled logistic regression, where predictors included covariate values at the time of waitlist registration (i.e., baseline covariates) to improve the stability of the weights.

Stabilized IPCW (${scw}_{i}$) were similarly constructed as the cumulative product of the probability of being censored at each day, conditional on individuals not having been censored or transplanted yet (observed through the prior day; $\bar{C}_{\left( k-1 \right)i}$ and $\bar{A}_{\left( k-1 \right)i}$, respectively) and covariate history observed through the current day ($\bar{L}_{\mathrm{ki}}$):

$${sw\_c}_{i}=\frac{\prod_{k=0}^{K} Pr[C_{\mathrm{ki}}=0|\bar{C}_{\left( k-1 \right)i}=\bar{0}, \bar{A}_{\left( k-1 \right)i}=\bar{0}]}{\prod_{k=0}^{K} Pr[C_{\mathrm{ki}}=0|\bar{C}_{\left( k-1 \right)i}=\bar{0}, \bar{A}_{\left( k-1 \right)i}=\bar{0}, \bar{L}_{\mathrm{ki}}=\bar{l}_{\mathrm{ki}}]}$$

These IPCW weights were obtained by fitting pooled logistic regression models with pre-transplant censoring (e.g., loss to follow-up or removal from the waitlist for other clinical reasons, such as being too sick to withstand the transplant surgery) as the outcome, and predictors including variables in the pre-transplant LAS, these same covariates lagged by one month, time spent on the waitlist, geography, gender, race, and height. The final, time-varying pre-transplant weight was then calculated as the product of the stabilized IPTW and stabilized IPCW at each time point: ${swscw}_{i}={sw}_{i}*{scw}_{i}$.

Among patients who received transplant, the denominator of the IPTW is the same as the denominator of the pre-transplant IPTW described above. The numerator, however, includes the transplant indicator only. Covariate values at waitlist registration were excluded from the numerator of the post-transplant IPTW because these covariates are not present in the post-transplant outcome model [4, 5]. The post-transplant IPTW was then taken to be the cumulative product of this marginally stabilized IPTW associated with the last record for each transplanted patient in the pre-transplant data set.

The post-transplant stabilized IPCW was estimated by fitting a logistic regression model with the post-transplant censoring variable (i.e., loss to follow-up after transplant) as the outcome and the covariates of the published post-transplant LAS as predictors (i.e., age, cardiac index, continuous mechanical ventilation, serum creatinine, diagnosis group, functional status, oxygen need at rest, six-minute walk distance), along with geography, gender, race, and time (days) spent on the waitlist (modeled using restricted cubic splines with five knots; knots were chosen using Harrell’s recommended percentiles (i.e., 5, 27.5, 50, 72.5, and 95), as implemented by STATA’s *mkspline* function [3]). The final post-transplant weight was calculated as the product of the stabilized IPTW and stabilized IPCW. To minimize the impact of extreme weights on our outcome models, we progressively truncated the final pre- and post-transplant weights following the procedure outlined in [6], with weights finally truncated at the 0.25% (99.75%) percentile. Distributions of these weights appear in Appendix 5.

# APPENDIX 5: WEIGHT TRUNCATION

To minimize the impact of extreme weights on our outcome models, we progressively truncated the final pre- and post-transplant weights following the procedure outlined in [6]. More specifically, we replaced weights that were below (above) a certain percentile with the value at that percentile. By progressively increasing (decreasing) the percentiles from 0% (100%) to 5% (95%), we could explore the trade-off between bias and variance. Appendix Table 3 displays the distribution of the pre-transplant weights, alternatively-stabilized pre-transplant weights (i.e., those used to construct the post-transplant weights; see main text for details), and post-transplant weights under various truncation percentiles. As the extent of truncation increases, the mean of the weights moves further away from one, indicating a greater degree of bias; conversely, the variance of the weights moves closer to zero, suggesting greater precision. Ultimately, the final weights were truncated at the 0.25% (99.75%) percentiles, as these were the ones which centered the weights around one and which reduced the 1/minimum and maximum weights by at least one order of magnitude [6].

**Appendix Table 3.** Distribution of pre- and post-transplant weights under various truncation percentiles. The distribution of the final weights appears in bold.

**Appendix Table 3A)** Distribution of pre-transplant weights

| **Truncation Percentiles** | **Mean** | **Variance** | **Minimum** | **Maximum** |
| --- | --- | --- | --- | --- |
| 0, 100 | 1.127 | 26.91 | 1.28E-09 | 551.0 |
| **0.25, 99.75** | **1.034** | **0.817** | **0.001** | **10.29** |
| 0.5, 99.5 | 1.024 | 0.666 | 0.003 | 7.386 |
| 1, 99 | 1.006 | 0.485 | 0.010 | 4.832 |
| 2.5, 97.5 | 0.976 | 0.316 | 0.042 | 2.902 |
| 3.5, 96.5 | 0.962 | 0.265 | 0.069 | 2.396 |
| 5, 95 | 0.948 | 0.223 | 0.113 | 2.025 |

**Appendix Table 3B)** Distribution of alternatively-stabilized pre-transplant weights

| **Truncation Percentiles** | **Mean** | **Variance** | **Minimum** | **Maximum** |
| --- | --- | --- | --- | --- |
| 0, 100 | 1.148 | 530.7 | 9.81E-05 | 1.01E+04 |
| **0.25, 99.75** | **0.959** | **1.492** | **0.001** | **14.88** |
| 0.5, 99.5 | 0.941 | 1.080 | 0.001 | 9.656 |
| 1, 99 | 0.915 | 0.729 | 0.003 | 6.020 |
| 2.5, 97.5 | 0.871 | 0.427 | 0.011 | 3.070 |
| 3.5, 96.5 | 0.856 | 0.370 | 0.017 | 2.572 |
| 5, 95 | 0.839 | 0.316 | 0.028 | 2.147 |

**Appendix Table 3C)** Distribution of post-transplant weights

| **Truncation Percentiles** | **Mean** | **Variance** | **Minimum** | **Maximum** |
| --- | --- | --- | --- | --- |
| 0, 100 | 1.175 | 75.33 | 1.50E-04 | 599.8 |
| **0.25, 99.75** | **0.962** | **1.625** | **0.009** | **17.09** |
| 0.5, 99.5 | 0.936 | 0.998 | 0.023 | 9.531 |
| 1, 99 | 0.905 | 0.621 | 0.033 | 4.948 |
| 2.5, 97.5 | 0.875 | 0.445 | 0.059 | 2.969 |
| 3.5, 96.5 | 0.862 | 0.394 | 0.077 | 2.503 |
| 5, 95 | 0.847 | 0.345 | 0.103 | 2.099 |

# APPENDIX 6: PARAMETER ESTIMATES FOR MODIFIED OUTCOME MODELS

Here, we display the parameter estimates (Appendix Table 4) and baseline survival probabilities (Appendix Table 5) obtained from the modified (weighted) pre- and post-transplant outcome models.

**Appendix Table 4.** Parameter estimates obtained from A) the modified (weighted) pre-transplant outcome model, and B) the modified (weighted) post-transplant outcome model.

**Appendix Table 4A)** Parameter estimates obtained from the modified pre-transplant outcome model

| Covariate | Coefficient Estimate | 95% Confidence Interval |
| --- | --- | --- |
| Age at offer (years) | -0.0024318 | -0.0113106, 0.0064469 |
| Bilirubin (mg/dL) | 0 | (omitted) |
| Bilirubin increase ≥50% | 0 | (omitted) |
| Body mass index (BMI) (kg/m^3^) | 0.0104932 | -0.0058752, 0.0268615 |
| Cardiac index (L/min/m^2^) | 0.4723494 | 0.2710321, 0.6736667 |
| Central venous pressure (CVP) (mmHg) | -0.0503087 | -0.1253766, 0.0247593 |
| Continuous mechanical ventilation | 1.22271 | 0.6709733, 1.774447 |
| Creatinine (serum) (mg/dL) | 0.0864787 | 0.0193925, 0.1535648 |
| Diabetes | 0.3591762 | 0.137798, 0.5805544 |
| Diagnosis Group A | REF |  |
| Diagnosis Group B | 0.665212 | 0.0004605, 1.329963 |
| Diagnosis Group C | 0.2546667 | -0.2330827, 0.7424161 |
| Diagnosis Group D | 0.8609124 | 0.3824339, 1.339391 |
| Forced vital capacity (FVC) % predicted | 0.0682059 | -0.0151386, 0.1515504 |
| Functional status (none) | 0.0379326 | -0.32105, 0.3969151 |
| Oxygen need at rest (L/min) | 0.0520477 | 0.024774, 0.0793213 |
| Oxygen-by-diagnosis interaction | 0.0301022 | -0.0152716, 0.0754759 |
| pCO_2_ | 0.1595511 | 0.0794078, 0.2396944 |
| pCO_2_ increase ≥15% | 0 | (omitted) |
| Pulmonary artery (PA) systolic pressure (mmHg) | 0.1009163 | 0.0482246, 0.153608 |
| PA-by-diagnosis interaction | 0.0040516 | -0.0967816, 0.1048847 |
| Six-minute walk distance (feet) | -0.0130285 | -0.0300434, 0.0039865 |

**Appendix Table 4B)** Parameter estimates obtained from the modified post-transplant outcome model

| Covariate | Coefficient Estimate | 95% Confidence Interval |
| --- | --- | --- |
| Age at transplant (years) | 0.0310272 | 0.0212575, 0.0407969 |
| Cardiac index (L/min/m^2^) | 0.1035634 | -0.0367848, 0.2439117 |
| Continuous mechanical ventilation | 0.5672613 | 0.1808594, 0.9536632 |
| Creatinine (serum) (mg/dL) | 0.2231819 | 0.0890585, 0.3573053 |
| Creatinine increase ≥150% | 0 | (omitted) |
| Diagnosis Group A | REF |  |
| Diagnosis Group B | 0.3578133 | 0.0197239, 0.6959027 |
| Diagnosis Group C | 0.16691 | -0.123965, 0.4577851 |
| Diagnosis Group D | 0.0524997 | -0.1283522, 0.2333516 |
| Functional status (none) | -0.2999765 | -0.5940731, -.00588 |
| Oxygen need at rest (L/min) | 0.0166301 | 0.0014614, 0.0317989 |
| Oxygen-by-diagnosis interaction | -0.0094877 | -0.039502, 0.0205267 |
| Six-minute walk distance (feet) | -0.0000709 | -0.0002148, 0.0000729 |

**Appendix Table 5.** Baseline survival probabilities obtained from A) the modified (weighted) pre-transplant outcome model, and B) the modified (weighted) post-transplant outcome model.

**Appendix Table 5A)** Baseline survival probabilities for the modified pre-transplant outcome model

| Time (days) | Waitlist Survival |
| --- | --- |
| 0 | 1.00000000 |
| 1 | 0.99949890 |
| 2 | 0.99861080 |
| 3 | 0.99847020 |
| 4 | 0.99824380 |
| 5 | 0.99809990 |
| 6 | 0.99787550 |
| 7 | 0.99771830 |
| 8 | 0.99762050 |
| 9 | 0.99752650 |
| 10 | 0.99738530 |
| 11 | 0.99721320 |
| 12 | 0.99715210 |
| 13 | 0.99700950 |
| 14 | 0.99690890 |
| 15 | 0.99680770 |
| 16 | 0.99673020 |
| 17 | 0.99669440 |
| 18 | 0.99663090 |
| 19 | 0.99651170 |
| 20 | 0.99637040 |
| 21 | 0.99633550 |
| 22 | 0.99630000 |
| 23 | 0.99622360 |
| 24 | 0.99618820 |
| 25 | 0.99607970 |
| 26 | 0.99606270 |
| 27 | 0.99595380 |
| 28 | 0.99588110 |
| 29 | 0.99580880 |
| 30 | 0.99580880 |
| 31 | 0.99575660 |
| 32 | 0.99575660 |
| 33 | 0.99555590 |
| 34 | 0.99543440 |
| 35 | 0.99535690 |
| 36 | 0.99529670 |
| 37 | 0.99504970 |
| 38 | 0.99498210 |
| 39 | 0.99482720 |
| 40 | 0.99478720 |
| 41 | 0.99470050 |
| 42 | 0.99467110 |
| 43 | 0.99464450 |
| 44 | 0.99457450 |
| 45 | 0.99455430 |
| 46 | 0.99449440 |
| 47 | 0.99449440 |
| 48 | 0.99439620 |
| 49 | 0.99436790 |
| 50 | 0.99429490 |
| 51 | 0.99427350 |
| 52 | 0.99413210 |
| 53 | 0.99409040 |
| 54 | 0.99397610 |
| 55 | 0.99387900 |
| 56 | 0.99387900 |
| 57 | 0.99361910 |
| 58 | 0.99361910 |
| 59 | 0.99353570 |
| 60 | 0.99351250 |
| 61 | 0.99343780 |
| 62 | 0.99334730 |
| 63 | 0.99327860 |
| 64 | 0.99303570 |
| 65 | 0.99298630 |
| 66 | 0.99294020 |
| 67 | 0.99294020 |
| 68 | 0.99278280 |
| 69 | 0.99270620 |
| 70 | 0.99225440 |
| 71 | 0.99217070 |
| 72 | 0.99211770 |
| 73 | 0.99205410 |
| 74 | 0.99195170 |
| 75 | 0.99192470 |
| 76 | 0.99188070 |
| 77 | 0.99188070 |
| 78 | 0.99183380 |
| 79 | 0.99180110 |
| 80 | 0.99174440 |
| 81 | 0.99174440 |
| 82 | 0.99171980 |
| 83 | 0.99169260 |
| 84 | 0.99169260 |
| 85 | 0.99165900 |
| 86 | 0.99161170 |
| 87 | 0.99157360 |
| 88 | 0.99144450 |
| 89 | 0.99127280 |
| 90 | 0.99127280 |
| 91 | 0.99127280 |
| 92 | 0.99123600 |
| 93 | 0.99105800 |
| 94 | 0.99097330 |
| 95 | 0.99069050 |
| 96 | 0.99065850 |
| 97 | 0.99057640 |
| 98 | 0.99057640 |
| 99 | 0.99057640 |
| 100 | 0.99057640 |
| 101 | 0.99057640 |
| 102 | 0.99050870 |
| 103 | 0.99045280 |
| 104 | 0.99045280 |
| 105 | 0.99042720 |
| 106 | 0.99032910 |
| 107 | 0.99032910 |
| 108 | 0.99032910 |
| 109 | 0.99032910 |
| 110 | 0.99028200 |
| 111 | 0.99028200 |
| 112 | 0.99025570 |
| 113 | 0.99022540 |
| 114 | 0.99015130 |
| 115 | 0.99009470 |
| 116 | 0.99009470 |
| 117 | 0.99009470 |
| 118 | 0.99009470 |
| 119 | 0.99004220 |
| 120 | 0.98999600 |
| 121 | 0.98999600 |
| 122 | 0.98990010 |
| 123 | 0.98990010 |
| 124 | 0.98987070 |
| 125 | 0.98987070 |
| 126 | 0.98979010 |
| 127 | 0.98975310 |
| 128 | 0.98970750 |
| 129 | 0.98957860 |
| 130 | 0.98945660 |
| 131 | 0.98945660 |
| 132 | 0.98945660 |
| 133 | 0.98938810 |
| 134 | 0.98927390 |
| 135 | 0.98927390 |
| 136 | 0.98924580 |
| 137 | 0.98924580 |
| 138 | 0.98924580 |
| 139 | 0.98901980 |
| 140 | 0.98898180 |
| 141 | 0.98898180 |
| 142 | 0.98891120 |
| 143 | 0.98881060 |
| 144 | 0.98881060 |
| 145 | 0.98881060 |
| 146 | 0.98862540 |
| 147 | 0.98862540 |
| 148 | 0.98859800 |
| 149 | 0.98859800 |
| 150 | 0.98855410 |
| 151 | 0.98855410 |
| 152 | 0.98850870 |
| 153 | 0.98846440 |
| 154 | 0.98846440 |
| 155 | 0.98838650 |
| 156 | 0.98833870 |
| 157 | 0.98833870 |
| 158 | 0.98833870 |
| 159 | 0.98828630 |
| 160 | 0.98828630 |
| 161 | 0.98810580 |
| 162 | 0.98810580 |
| 163 | 0.98807000 |
| 164 | 0.98807000 |
| 165 | 0.98804610 |
| 166 | 0.98799710 |
| 167 | 0.98799710 |
| 168 | 0.98799710 |
| 169 | 0.98799710 |
| 170 | 0.98792760 |
| 171 | 0.98789130 |
| 172 | 0.98784470 |
| 173 | 0.98784470 |
| 174 | 0.98778040 |
| 175 | 0.98778040 |
| 176 | 0.98762660 |
| 177 | 0.98752250 |
| 178 | 0.98752250 |
| 179 | 0.98752250 |
| 180 | 0.98752250 |
| 181 | 0.98747760 |
| 182 | 0.98686000 |
| 183 | 0.98643590 |
| 184 | 0.98606400 |
| 185 | 0.98558710 |
| 186 | 0.98555720 |
| 187 | 0.98547530 |
| 188 | 0.98542620 |
| 189 | 0.98535930 |
| 190 | 0.98530210 |
| 191 | 0.98530210 |
| 192 | 0.98530210 |
| 193 | 0.98517610 |
| 194 | 0.98517610 |
| 195 | 0.98517610 |
| 196 | 0.98517610 |
| 197 | 0.98498220 |
| 198 | 0.98489450 |
| 199 | 0.98480400 |
| 200 | 0.98465610 |
| 201 | 0.98465610 |
| 202 | 0.98465610 |
| 203 | 0.98457030 |
| 204 | 0.98457030 |
| 205 | 0.98457030 |
| 206 | 0.98452710 |
| 207 | 0.98444500 |
| 208 | 0.98444500 |
| 209 | 0.98440380 |
| 210 | 0.98440380 |
| 211 | 0.98440380 |
| 212 | 0.98440380 |
| 213 | 0.98440380 |
| 214 | 0.98422070 |
| 215 | 0.98422070 |
| 216 | 0.98406430 |
| 217 | 0.98406430 |
| 218 | 0.98406430 |
| 219 | 0.98398360 |
| 220 | 0.98398360 |
| 221 | 0.98398360 |
| 222 | 0.98398360 |
| 223 | 0.98398360 |
| 224 | 0.98392360 |
| 225 | 0.98384980 |
| 226 | 0.98378170 |
| 227 | 0.98378170 |
| 228 | 0.98378170 |
| 229 | 0.98353850 |
| 230 | 0.98353850 |
| 231 | 0.98329040 |
| 232 | 0.98317210 |
| 233 | 0.98310490 |
| 234 | 0.98304650 |
| 235 | 0.98304650 |
| 236 | 0.98304650 |
| 237 | 0.98304650 |
| 238 | 0.98300730 |
| 239 | 0.98295540 |
| 240 | 0.98242220 |
| 241 | 0.98236150 |
| 242 | 0.98229290 |
| 243 | 0.98229290 |
| 244 | 0.98229290 |
| 245 | 0.98229290 |
| 246 | 0.98229290 |
| 247 | 0.98229290 |
| 248 | 0.98217620 |
| 249 | 0.98217620 |
| 250 | 0.98217620 |
| 251 | 0.98202600 |
| 252 | 0.98202600 |
| 253 | 0.98202600 |
| 254 | 0.98202600 |
| 255 | 0.98202600 |
| 256 | 0.98195530 |
| 257 | 0.98185160 |
| 258 | 0.98185160 |
| 259 | 0.98176630 |
| 260 | 0.98176630 |
| 261 | 0.98139880 |
| 262 | 0.98131640 |
| 263 | 0.98125940 |
| 264 | 0.98125940 |
| 265 | 0.98125940 |
| 266 | 0.98104180 |
| 267 | 0.98090980 |
| 268 | 0.98080010 |
| 269 | 0.98080010 |
| 270 | 0.98039670 |
| 271 | 0.98032500 |
| 272 | 0.98032500 |
| 273 | 0.98032500 |
| 274 | 0.98032500 |
| 275 | 0.98032500 |
| 276 | 0.98021850 |
| 277 | 0.98001860 |
| 278 | 0.98001860 |
| 279 | 0.97993420 |
| 280 | 0.97993420 |
| 281 | 0.97985390 |
| 282 | 0.97985390 |
| 283 | 0.97985390 |
| 284 | 0.97985390 |
| 285 | 0.97985390 |
| 286 | 0.97985390 |
| 287 | 0.97985390 |
| 288 | 0.97976220 |
| 289 | 0.97976220 |
| 290 | 0.97976220 |
| 291 | 0.97976220 |
| 292 | 0.97975350 |
| 293 | 0.97975350 |
| 294 | 0.97963670 |
| 295 | 0.97954550 |
| 296 | 0.97954550 |
| 297 | 0.97954550 |
| 298 | 0.97939460 |
| 299 | 0.97930350 |
| 300 | 0.97930350 |
| 301 | 0.97930350 |
| 302 | 0.97920520 |
| 303 | 0.97912270 |
| 304 | 0.97912270 |
| 305 | 0.97912270 |
| 306 | 0.97908000 |
| 307 | 0.97908000 |
| 308 | 0.97908000 |
| 309 | 0.97908000 |
| 310 | 0.97908000 |
| 311 | 0.97896840 |
| 312 | 0.97896840 |
| 313 | 0.97896840 |
| 314 | 0.97896840 |
| 315 | 0.97896840 |
| 316 | 0.97896840 |
| 317 | 0.97894650 |
| 318 | 0.97894650 |
| 319 | 0.97881190 |
| 320 | 0.97881190 |
| 321 | 0.97874950 |
| 322 | 0.97874950 |
| 323 | 0.97874950 |
| 324 | 0.97874950 |
| 325 | 0.97874950 |
| 326 | 0.97874950 |
| 327 | 0.97874950 |
| 328 | 0.97874950 |
| 329 | 0.97874950 |
| 330 | 0.97874950 |
| 331 | 0.97874950 |
| 332 | 0.97874950 |
| 333 | 0.97868070 |
| 334 | 0.97868070 |
| 335 | 0.97868070 |
| 336 | 0.97868070 |
| 337 | 0.97854430 |
| 338 | 0.97848880 |
| 339 | 0.97848880 |
| 340 | 0.97807370 |
| 341 | 0.97807370 |
| 342 | 0.97807370 |
| 343 | 0.97801020 |
| 344 | 0.97766270 |
| 345 | 0.97766270 |
| 346 | 0.97766270 |
| 347 | 0.97766270 |
| 348 | 0.97761010 |
| 349 | 0.97761010 |
| 350 | 0.97761010 |
| 351 | 0.97761010 |
| 352 | 0.97758430 |
| 353 | 0.97737180 |
| 354 | 0.97737180 |
| 355 | 0.97737180 |
| 356 | 0.97721110 |
| 357 | 0.97721110 |
| 358 | 0.97721110 |
| 359 | 0.97721110 |
| 360 | 0.97705880 |
| 361 | 0.97705880 |
| 362 | 0.97705880 |
| 363 | 0.97705880 |
| 364 | 0.97705880 |

**Appendix Table 5B)** Baseline survival probabilities for the modified post-transplant outcome model

| Time (days) | Post-transplant Survival |
| --- | --- |
| 0 | 0.998379000 |
| 1 | 0.996209000 |
| 2 | 0.995379400 |
| 3 | 0.994953900 |
| 4 | 0.993997900 |
| 5 | 0.992298200 |
| 6 | 0.991685700 |
| 7 | 0.991396500 |
| 8 | 0.991039700 |
| 9 | 0.990595300 |
| 10 | 0.989299400 |
| 11 | 0.988702900 |
| 12 | 0.987841900 |
| 13 | 0.987356600 |
| 14 | 0.987100000 |
| 15 | 0.986487900 |
| 16 | 0.986323600 |
| 17 | 0.986016400 |
| 18 | 0.985647500 |
| 19 | 0.985276200 |
| 20 | 0.984772100 |
| 21 | 0.984616200 |
| 22 | 0.984040200 |
| 23 | 0.983644800 |
| 24 | 0.982689800 |
| 25 | 0.981989400 |
| 26 | 0.980997600 |
| 27 | 0.980587300 |
| 28 | 0.980077000 |
| 29 | 0.979670700 |
| 30 | 0.979599900 |
| 31 | 0.979448300 |
| 32 | 0.979286400 |
| 33 | 0.979130000 |
| 34 | 0.978923600 |
| 35 | 0.978855600 |
| 36 | 0.978692100 |
| 37 | 0.978400000 |
| 38 | 0.978172400 |
| 39 | 0.977904100 |
| 40 | 0.977776900 |
| 41 | 0.977734100 |
| 42 | 0.977639500 |
| 43 | 0.977488500 |
| 44 | 0.977392400 |
| 45 | 0.977168300 |
| 46 | 0.977003700 |
| 47 | 0.976958500 |
| 48 | 0.976732100 |
| 49 | 0.976600200 |
| 50 | 0.976471600 |
| 51 | 0.976109700 |
| 52 | 0.976018100 |
| 53 | 0.975722700 |
| 54 | 0.974344400 |
| 55 | 0.973900600 |
| 56 | 0.973895700 |
| 57 | 0.973762800 |
| 58 | 0.973460100 |
| 59 | 0.973216100 |
| 60 | 0.973103000 |
| 61 | 0.973052600 |
| 62 | 0.972896600 |
| 63 | 0.972679700 |
| 64 | 0.972528200 |
| 65 | 0.972342200 |
| 66 | 0.972271300 |
| 67 | 0.972008500 |
| 68 | 0.971873800 |
| 69 | 0.971872000 |
| 70 | 0.971683500 |
| 71 | 0.971650800 |
| 72 | 0.970162600 |
| 73 | 0.970044900 |
| 74 | 0.969789700 |
| 75 | 0.969757400 |
| 76 | 0.969443500 |
| 77 | 0.969000000 |
| 78 | 0.968453000 |
| 79 | 0.967538600 |
| 80 | 0.967524800 |
| 81 | 0.967168700 |
| 82 | 0.966833000 |
| 83 | 0.966816600 |
| 84 | 0.966757400 |
| 85 | 0.966510500 |
| 86 | 0.966219100 |
| 87 | 0.965916700 |
| 88 | 0.965883700 |
| 89 | 0.965768100 |
| 90 | 0.965748000 |
| 91 | 0.965563800 |
| 92 | 0.965503500 |
| 93 | 0.965338700 |
| 94 | 0.965007600 |
| 95 | 0.964726900 |
| 96 | 0.964699200 |
| 97 | 0.964074500 |
| 98 | 0.963914900 |
| 99 | 0.963844300 |
| 100 | 0.963640400 |
| 101 | 0.963522400 |
| 102 | 0.963408300 |
| 103 | 0.963045300 |
| 104 | 0.962898400 |
| 105 | 0.962898400 |
| 106 | 0.962100800 |
| 107 | 0.962046800 |
| 108 | 0.962046800 |
| 109 | 0.961838900 |
| 110 | 0.961772900 |
| 111 | 0.961755900 |
| 112 | 0.961686100 |
| 113 | 0.961529700 |
| 114 | 0.961294400 |
| 115 | 0.961167500 |
| 116 | 0.961068800 |
| 117 | 0.961046200 |
| 118 | 0.960706400 |
| 119 | 0.960585500 |
| 120 | 0.960303000 |
| 121 | 0.960256100 |
| 122 | 0.960174400 |
| 123 | 0.959945900 |
| 124 | 0.959896700 |
| 125 | 0.959729300 |
| 126 | 0.959527900 |
| 127 | 0.959465300 |
| 128 | 0.958906800 |
| 129 | 0.958779200 |
| 130 | 0.957531500 |
| 131 | 0.957405300 |
| 132 | 0.957405300 |
| 133 | 0.957353700 |
| 134 | 0.957239700 |
| 135 | 0.957239700 |
| 136 | 0.957233100 |
| 137 | 0.957141500 |
| 138 | 0.957141500 |
| 139 | 0.957070300 |
| 140 | 0.956980500 |
| 141 | 0.956749500 |
| 142 | 0.956740300 |
| 143 | 0.956274000 |
| 144 | 0.956183900 |
| 145 | 0.956071300 |
| 146 | 0.955805500 |
| 147 | 0.955735200 |
| 148 | 0.955626400 |
| 149 | 0.955425500 |
| 150 | 0.955280800 |
| 151 | 0.955147300 |
| 152 | 0.955049300 |
| 153 | 0.954756700 |
| 154 | 0.954693900 |
| 155 | 0.954568900 |
| 156 | 0.954542000 |
| 157 | 0.954471600 |
| 158 | 0.954321000 |
| 159 | 0.954193400 |
| 160 | 0.954054100 |
| 161 | 0.954054100 |
| 162 | 0.953888700 |
| 163 | 0.953675300 |
| 164 | 0.953573600 |
| 165 | 0.953506300 |
| 166 | 0.953472300 |
| 167 | 0.953452700 |
| 168 | 0.953325100 |
| 169 | 0.953237900 |
| 170 | 0.953237900 |
| 171 | 0.952996100 |
| 172 | 0.952936600 |
| 173 | 0.952710200 |
| 174 | 0.952282200 |
| 175 | 0.952176300 |
| 176 | 0.952116600 |
| 177 | 0.952017100 |
| 178 | 0.951952000 |
| 179 | 0.951952000 |
| 180 | 0.951952000 |
| 181 | 0.951950000 |
| 182 | 0.951911600 |
| 183 | 0.951601600 |
| 184 | 0.951466600 |
| 185 | 0.951463200 |
| 186 | 0.951400900 |
| 187 | 0.951214000 |
| 188 | 0.951092900 |
| 189 | 0.950993600 |
| 190 | 0.950847200 |
| 191 | 0.950584600 |
| 192 | 0.950504200 |
| 193 | 0.950463300 |
| 194 | 0.950147200 |
| 195 | 0.950147200 |
| 196 | 0.949776700 |
| 197 | 0.949665600 |
| 198 | 0.949574900 |
| 199 | 0.949529500 |
| 200 | 0.949529500 |
| 201 | 0.949463900 |
| 202 | 0.949422900 |
| 203 | 0.949243500 |
| 204 | 0.949027900 |
| 205 | 0.948980500 |
| 206 | 0.948826800 |
| 207 | 0.948815700 |
| 208 | 0.948689100 |
| 209 | 0.948545700 |
| 210 | 0.948230000 |
| 211 | 0.948183900 |
| 212 | 0.948036900 |
| 213 | 0.947931400 |
| 214 | 0.947726400 |
| 215 | 0.947523600 |
| 216 | 0.947122800 |
| 217 | 0.946956200 |
| 218 | 0.946857200 |
| 219 | 0.946711000 |
| 220 | 0.946613100 |
| 221 | 0.946086500 |
| 222 | 0.945987800 |
| 223 | 0.945961600 |
| 224 | 0.945961600 |
| 225 | 0.945942200 |
| 226 | 0.945703000 |
| 227 | 0.945703000 |
| 228 | 0.945670900 |
| 229 | 0.945435400 |
| 230 | 0.945435400 |
| 231 | 0.945277600 |
| 232 | 0.945208200 |
| 233 | 0.945014400 |
| 234 | 0.944705400 |
| 235 | 0.944600100 |
| 236 | 0.944469400 |
| 237 | 0.944377700 |
| 238 | 0.944296600 |
| 239 | 0.944296600 |
| 240 | 0.943992600 |
| 241 | 0.943724900 |
| 242 | 0.943714900 |
| 243 | 0.943588400 |
| 244 | 0.943588400 |
| 245 | 0.943468900 |
| 246 | 0.943468900 |
| 247 | 0.943394300 |
| 248 | 0.943310700 |
| 249 | 0.943023900 |
| 250 | 0.942614300 |
| 251 | 0.942614300 |
| 252 | 0.942598800 |
| 253 | 0.942598800 |
| 254 | 0.942598800 |
| 255 | 0.942313600 |
| 256 | 0.942255900 |
| 257 | 0.942201500 |
| 258 | 0.942201500 |
| 259 | 0.942201500 |
| 260 | 0.941973900 |
| 261 | 0.941789600 |
| 262 | 0.941781600 |
| 263 | 0.941551900 |
| 264 | 0.941510100 |
| 265 | 0.941169200 |
| 266 | 0.941169200 |
| 267 | 0.940625100 |
| 268 | 0.940375000 |
| 269 | 0.940290800 |
| 270 | 0.940290800 |
| 271 | 0.940124700 |
| 272 | 0.939783100 |
| 273 | 0.939713600 |
| 274 | 0.939536700 |
| 275 | 0.939360300 |
| 276 | 0.939360300 |
| 277 | 0.939224500 |
| 278 | 0.939219000 |
| 279 | 0.939219000 |
| 280 | 0.939210000 |
| 281 | 0.938749000 |
| 282 | 0.938478000 |
| 283 | 0.938264200 |
| 284 | 0.937949200 |
| 285 | 0.937949200 |
| 286 | 0.937933700 |
| 287 | 0.937672600 |
| 288 | 0.937491100 |
| 289 | 0.937464300 |
| 290 | 0.937374400 |
| 291 | 0.937180800 |
| 292 | 0.937130700 |
| 293 | 0.936952600 |
| 294 | 0.936940400 |
| 295 | 0.936704100 |
| 296 | 0.936677300 |
| 297 | 0.936576600 |
| 298 | 0.936576600 |
| 299 | 0.936544200 |
| 300 | 0.936416600 |
| 301 | 0.936385300 |
| 302 | 0.936285700 |
| 303 | 0.936217100 |
| 304 | 0.936217100 |
| 305 | 0.936097700 |
| 306 | 0.936017000 |
| 307 | 0.935961000 |
| 308 | 0.935924900 |
| 309 | 0.935584800 |
| 310 | 0.935510100 |
| 311 | 0.935276100 |
| 312 | 0.935252500 |
| 313 | 0.935242800 |
| 314 | 0.935242800 |
| 315 | 0.935234600 |
| 316 | 0.935234600 |
| 317 | 0.935234600 |
| 318 | 0.935081600 |
| 319 | 0.935081600 |
| 320 | 0.935081600 |
| 321 | 0.934945400 |
| 322 | 0.934927100 |
| 323 | 0.934898800 |
| 324 | 0.934809200 |
| 325 | 0.934624700 |
| 326 | 0.934624700 |
| 327 | 0.934587700 |
| 328 | 0.934535600 |
| 329 | 0.934449000 |
| 330 | 0.934449000 |
| 331 | 0.934414200 |
| 332 | 0.934267800 |
| 333 | 0.933950700 |
| 334 | 0.933911100 |
| 335 | 0.933706200 |
| 336 | 0.933689000 |
| 337 | 0.933689000 |
| 338 | 0.933604400 |
| 339 | 0.933426600 |
| 340 | 0.933280600 |
| 341 | 0.933167000 |
| 342 | 0.932964300 |
| 343 | 0.932928200 |
| 344 | 0.932928200 |
| 345 | 0.932928200 |
| 346 | 0.932729000 |
| 347 | 0.932729000 |
| 348 | 0.932472400 |
| 349 | 0.932472400 |
| 350 | 0.932273800 |
| 351 | 0.932266900 |
| 352 | 0.932266900 |
| 353 | 0.932230800 |
| 354 | 0.932166200 |
| 355 | 0.932071200 |
| 356 | 0.931930800 |
| 357 | 0.931914700 |
| 358 | 0.931643900 |
| 359 | 0.931368300 |
| 360 | 0.931017800 |
| 361 | 0.931009700 |
| 362 | 0.931009700 |
| 363 | 0.930992900 |
| 364 | 0.930986600 |

# APPENDIX 7: CALCULATING THE MODIFIED LAS SCORE

Modified LAS scores were calculated for each patient in our testing cohort by applying the weighted pre- and post-transplant outcome models to this cohort, considering all possible offer dates in 2016 and 2017. At each offer date, we subset the data to include only patients who were alive, registered on the waitlist, and not yet transplanted at that date. Then, we computed modified waitlist urgency and modified post-transplant survival measures following UNOS guidelines [1, 7]. We computed daily, person-specific survival estimates for the first year spent on the waitlist and the first year post-transplant using the baseline hazard, weighted model coefficients, and each individual’s covariate values. Each patients’ resulting waitlist and post-transplant survival probabilities were summed to obtain the modified waitlist urgency (${mWL}_{i}$) and modified post-transplant survival (${mPT}_{i}$) measures:

$${mWait}_{i}=\sum_{k=1}^{365} S_{Wait,(k-1)i}*1 day$$

$${mPT}_{i}=\sum_{k=1}^{365} S_{PT,(k-1)i}*1 day$$

where $S_{Wait,(k-1)i}$ and $S_{TX,(k-1)i}$ respectively represent the pre-transplant (waitlist) and post-transplant survival probabilities for subject $i$ at day $k-1$. The modified raw score was then computed as:

$$Modified Raw Score={mPT}_{i}-2*{mWait}_{i}$$

Taking into account the maximum and minimum pre- and post-transplant survival, the modified raw score was normalized via the following equation, consistent with the existing LAS:

$$Modified LAS=\frac{100*[\left( {Modified Raw Score}_{i} \right)+730]}{1095}$$

# APPENDIX 8: LONG-TERM CALIBRATION OF POST-TRANSPLANT OUTCOME MODEL

In the main text, we displayed time-dependent calibration plots for the first two years after transplant. However, recent literature suggests that longer-term post-transplant survival is of considerable interest to the lung transplant community [8-10]. Consequently, in this appendix we display time-dependent calibration plots for both the modified and existing post-transplant outcome models during the first eight years after transplant (Appendix Figure 2). This timeframe represents the full extent of follow-up time to which we have access for the development cohort. In the testing cohort, only two years of follow-up are available; thus, readers are referred back to Figure 2 (main text).

**
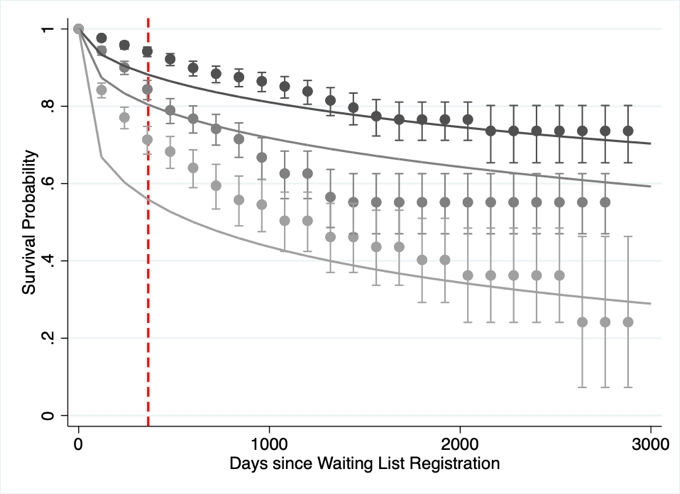

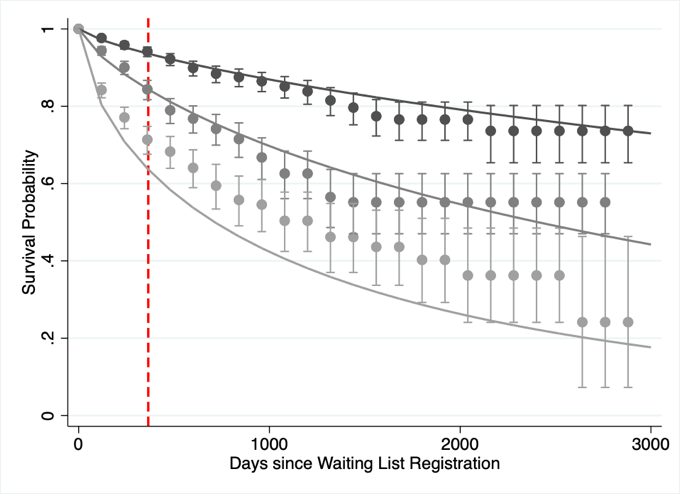
**

**A** **B**


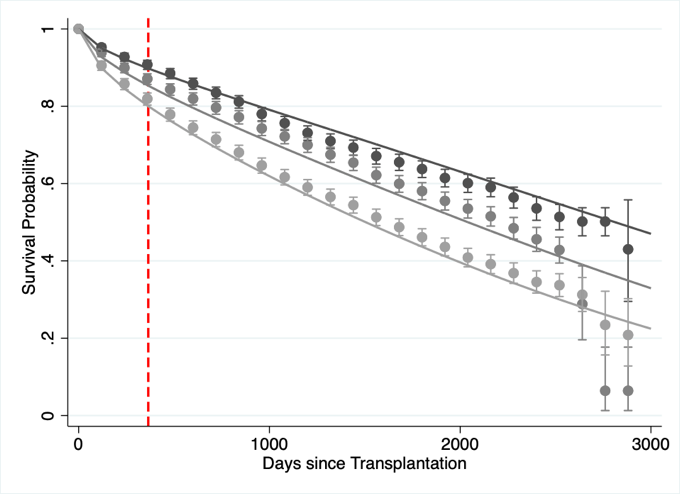

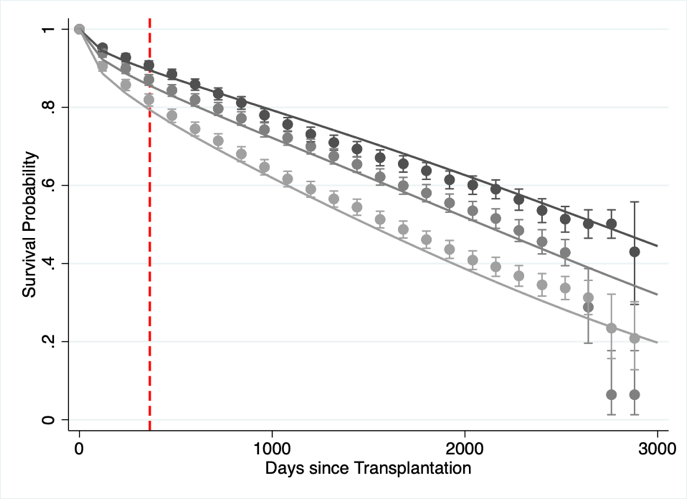


**C D**

**Appendix Figure 2. Observed vs. Predicted Survival in Development Cohort.** Time-dependent calibration of A) the modified pre-transplant outcome model, B) the existing pre-transplant LAS model, C) the modified post-transplant outcome model, and D) the existing post- transplant LAS model, in the development cohort. Smooth, solid lines represent predicted survival probabilities; points with vertical error bars represent observed Kaplan-Meier estimates with their corresponding 95% confidence intervals. Estimates were plotted every 30 days to ease plot readability. Three risk groups are shown: low-risk/best survival (darkest lines), medium-risk/intermediate survival (medium-shaded lines), and high-risk/worst survival (lightest lines). A vertical, dashed, red line is placed at one year post-waitlist registration for reference.

# REFERENCES

1. Organ Procurement and Transplantation Network (OPTN) Policies, effective 1 March 2020. Available: <<https://optn.transplant.hrsa.gov/media/1200/optn_policies.pdf>>. Accessed 10 March 2020.

2. Xiang, F. and S. Murray, *Restricted mean models for transplant benefit and urgency.* Stat Med, 2012. **31**(6): p. 561-76.

3. Harrell, F.J., *Regression Modeling Strategies: With Applications to Linear Models, Logistic Regression, and Survival Analysis*. 2001, New York: Springer.

4. Yang, W. and M.M. Joffe, *Subtle issues in model specification and estimation of marginal structural models.* Pharmacoepidemiol Drug Saf, 2012. **21**(3): p. 241-5.

5. Robins, J.M., M.A. Hernán, and B. Brumback, *Marginal structural models and causal inference in epidemiology.* Epidemiology, 2000. **11**(5): p. 550-60.

6. Cole, S.R. and M.A. Hernán, *Constructing inverse probability weights for marginal structural models.* Am J Epidemiol, 2008. **168**(6): p. 656-64.

7. United Network for Organ Sharing (2015). A Guide to Calculating the Lung Allocation Score. Available: <https://www.unos.org/wp-content/uploads/unos/lung_allocation_score.pdf>. Accessed 15 June 2018.

8. Maxwell, B.G., et al., *Impact of the lung allocation score on survival beyond 1 year.* Am J Transplant, 2014. **14**(10): p. 2288-94.

9. U.S. Department of Health & Human Services, Organ Procurement and Transplantation Network. Continuous Distribution. Available: <https://optn.transplant.hrsa.gov/governance/policy-initiatives/continuous-distribution/>. Accessed 6 October 2020.

10. Kasiske, B.L., J. Pyke, and J.J. Snyder, *Continuous distribution as an organ allocation framework.* Curr Opin Organ Transplant, 2020. **25**(2): p. 115-121.
